# Volatile metabolomics combined with rOAV and sensory evaluation reveals the aroma basis of black tea processed from the novel tea line ‘Jinlong No.1’ and its progenitor ‘Huangdan’

**DOI:** 10.3389/fpls.2026.1803743

**Published:** 2026-04-22

**Authors:** Jun Sun, Zhikun Lin, Liugang Zhu, Wenjin Zhang, Feng Wang

**Affiliations:** 1 Tea Research Institute, Fujian Academy of Agricultural Sciences, Fuzhou, China; 2 Agricultural Sciences Research Institute of Fuzhou, Fuzhou, China

**Keywords:** fruity-floral black tea, GC-MS/MS, ‘Jinlong No.1’, rOAV (relative odor activity value), sensory evaluation, tea processing, volatilome

## Abstract

Fruity-floral black tea quality is co-determined by both cultivar and processing. Jinlong No. 1 (JL1), a newly released high-aroma cultivar, lacked identified quantitative aroma markers. To elucidate the aroma characteristics of the newly released high-aroma cultivar ‘Jinlong No.1’ (JL1), three black teas were analyzed through GC-MS/MS-based volatile metabolomics, sensory evaluation, and relative odor activity value analysis. These included JL1 fruity-floral black tea (JL1B), JL1 conventional black tea (JL1S), and ‘Huangdan’ fruity-floral black tea (HDB). JL1B achieved the highest overall aroma score (8.2 ± 0.8). Comparative analysis revealed 340 differential volatiles between the two fruity-floral teas, predominantly esters (18%) and ketones (16%). Key upregulated compounds in JL1B included hexyl butanoate and hexyl 2-methylbutanoate, while (*E*)-2-nonenal and 1-nonen-3-ol were notably downregulated. Further comparison between the two JL1 teas identified 536 differential volatiles, with terpenoids (20%) and esters (18%) as the most abundant classes. Seventeen metabolites were commonly altered across comparisons, including the aforementioned esters. These findings establish specific quantitative targets for the precision processing and cultivar-directed breeding of premium fruity-floral black teas.

## 1 Introduction

Black tea is a globally popular beverage whose market value and consumer appeal are largely determined by its distinctive sensory characteristics, particularly aroma and taste (Ho et al., 2015). Black tea is characterized by its signature sweet and malty notes, which develop through enzymatic oxidation (fermentation) and thermal reactions during manufacturing. Beyond sensory aroma quality, black tea and its bioactive constituents have been associated with multiple health benefits. Regular consumption is linked to improved cardiovascular function, enhanced antioxidant capacity, and modulated metabolic homeostasis, primarily attributed to its abundant polyphenols such as catechins, theaflavins, and epigallocatechin gallate (EGCG) (Zhang et al., 2025). Recent studies further reported that EGCG derivatives possess antitumor and antioxidant activities (Paonessa et al., 2018), while tea polyphenols exert their protective effects through pivotal mechanisms involving free-radical scavenging and modulation of cell signaling pathways (Yan et al., 2020). Consequently, integrating these recognized health-promoting properties with cultivar-directed flavor innovation could potentially enhance both the sensory experience and functional value of fruity-floral black teas.

In recent years, innovation in black tea products has continuously expanded the boundaries of its flavor profile. Among these developments, fruity-floral black tea has emerged as an important direction. This type of black tea often draws upon processing techniques from other tea categories, particularly the shaking or tumbling methods used during oolong tea production. By applying mechanical stress to the tea leaves, these techniques promote the formation of unique volatile compounds, resulting in greater aromatic complexity and more pronounced top notes compared to traditional black teas.

High-aroma tea cultivars, primarily derived from oolong tea germplasm and their hybrid progenies, are considered ideal raw materials for producing high-quality fruity-floral black teas. Notable examples include Jinmudan, Jinguanyin, and several other high-aroma cultivars developed from ‘Huangdan’ and Tieguanyin lineages (Luo et al., 2017; Xu et al., 2024). ‘Huangdan’ (‘Huangjingui’) and ‘Jinguanyin’, two renowned high-aroma cultivars native to Fujian Province, have established reputations for yielding superior black tea with pronounced fruity-floral attributes. ‘Jinlong No. 1’ (JL1) is a hybrid tea line derived from Jinguanyin, which itself originated from ‘Huangdan’. When processed into black tea, JL1 exhibited even more prominent fruity-floral characteristics. This line also offered high yield and strong adaptability: the oolong tea made from it has a mellow taste and rich floral aroma, while its black tea counterpart presented a sweet, mellow flavor accompanied by distinct floral notes. However, despite extensive volatile compound studies on ‘Huangdan’ (You et al., 2025), JL1-specific quantitative aroma markers remain largely unidentified. Current volatile research emphasizes processing techniques—withering (Shan et al., 2022), fermentation (Gong et al., 2021), drying (Ye et al., 2021)—and geographical effects, but neglects systematic comparison between fruity-floral and traditional Congou black teas, particularly between JL1 and its ‘Huangdan’ progenitor. The integration of modern analytical techniques, particularly headspace solid-phase microextraction coupled with gas chromatography-mass spectrometry (HS-SPME-GC-MS), odor activity value (OAV) analysis, and multivariate statistical methods, provides a powerful toolkit for systematically deciphering key aroma compounds and flavor differences in black tea (Luo et al., 2017).

This study examines how cultivar (‘Jinlong No. 1’ vs. ‘Huangdan’) and processing (traditional vs. shaking-treated) independently and interactively affect black tea’s volatile metabolome and sensory quality. Using multi-factorial design with integrated GC-MS/MS and sensory analysis, we aim to clarify genetic and processing contributions to fruity-floral aroma and identify key odorants, establishing a scientific basis for optimized material selection and processing in premium black tea production.

## 2 Materials and methods

### 2.1 Experimental materials

The experimental materials consisted of fruity-floral black tea produced from the ‘Jinlong No.1’ cultivar and ‘Huangdan’ cultivar (samples JL1B and HDB) and traditional black tea produced from the ‘Jinlong No.1’ cultivar (sample JL1S). The plant materials of *Camellia sinensis* cv. ‘Jinlong No.1’ and ‘Huangdan’ were authenticated and provided by the Tea Research Institute of Fujian Academy of Agricultural Sciences in April 2024. Traditional black tea (JL1S) was produced from one bud and two leaves following standard congou processing: withering, rolling, fermentation, and drying. For the floral-style teas (JL1B and HDB), shoots of one bud and three-to-four leaves were harvested; after withering, the leaves received two successive *yaoqing* (gentle shaking) treatments (first: 2 min at 20 rpm; second: 8 min at 30 rpm) with a 2-h interval under controlled conditions (25 ± 2 °C, 80 ± 5% RH) before rolling, then completed the conventional rolling-fermentation-drying sequence of congou black-tea processing. All teas were produced in three independent batches (triplicates) to provide biological replication. The finished products were stored in airtight containers under dark and dry conditions. For analysis, samples were rapidly frozen using liquid nitrogen and ground into a fine powder using a ball mill (Retsch MM400). The powder was sieved through a 60-mesh screen and stored at −80 °C until further use.

### 2.2 Instruments and reagents

Volatile compounds were analyzed using a Gas Chromatography-Tandem Mass Spectrometry (GC-MS/MS) system (Agilent 8890-7000D, Agilent Technologies, Santa Clara, CA, USA) equipped with a DB-5MS capillary column (30 m × 0.25 mm × 0.25 μm, Agilent Technologies, Santa Clara, CA, USA). Solid-phase microextraction was performed using a 120 μm DVB/CWR/PDMS SPME Arrow fiber (Agilent Technologies, Santa Clara, CA, USA). Other key instruments included a Retsch MM400 ball mill (Retsch, Haan, Germany), a METTLER TOLEDO MS105DU analytical balance (Mettler-Toledo GmbH, Greifensee, Switzerland), and a CTC Analytics AG SPME Arrow autosampler system comprising a fiber conditioning station and an agitator sample heater (CTC Analytics, Zwingen, Switzerland).

The reagents used were sodium chloride (analytical grade, Sinopharm Group, Shanghai, China), n-hexane (chromatographic grade, Merck, Darmstadt, Germany), and 3-hexanone-2,2,4,4-d_4_ (Chromatographic grade, CDN Isotopes, Pointe-Claire, QC, Canada).

### 2.3 Extraction of volatile compounds

Frozen tea powder samples were removed from the −80 °C freezer. The quality control (QC) sample was prepared by pooling equal masses of all tested samples to monitor the analytical stability throughout the sequence. Following grinding in liquid nitrogen and vortex mixing, an aliquot of 500 mg was accurately weighed and transferred into a 20 mL headspace vial. A saturated NaCl solution and 20 µL of an internal standard solution (3-hexanone-2,2,4,4-d_4_, 10 µg/mL in n-hexane) were added to the vial for semi-quantification. The volatile compounds were then extracted using a fully automated Headspace Solid-Phase Microextraction (HS-SPME) system for subsequent GC-MS/MS analysis, as described by Ho et al. (2015) with modifications.

### 2.4 GC-MS/MS analysis conditions

HS-SPME extraction was conducted under the following conditions: sample incubation at 60 °C with agitation for 5 min, followed by extraction with the 120 μm DVB/CWR/PDMS fiber for 15 min at 60 °C, and final desorption in the GC injector port at 250 °C for 5 min. New fibers were conditioned at 250 °C for 2 h prior to initial use. The SPME Arrow system was employed to enhance extraction sensitivity.

Chromatographic separation was achieved using a DB-5MS capillary column with high-purity helium (≥99.999%) as the carrier gas at a constant flow rate of 1.2 mL/min. The injector temperature was set to 250 °C in splitless mode. The oven temperature program was as follows: initial hold at 40 °C for 3.5 min, ramped to 100 °C at 10 °C/min, then to 180 °C at 7 °C/min, finally to 280 °C at 25 °C/min, and held for 5 min. The solvent delay was set at 3.5 min.

Mass spectrometric detection was performed using an electron ionization (EI) source at 70 eV. The ion source temperature was maintained at 230 °C, the quadrupole temperature at 150 °C, and the transfer line temperature at 280 °C. Data acquisition was conducted in Selected-Ion-Monitoring (SIM) mode for targeted quantification, as detailed in Yuan et al. (2022) and Yuan et al. (2024).

### 2.5 Sensory evaluation

A trained sensory panel of 5 qualified tea tasters, selected and calibrated in accordance with GB/T 46555-2025, conducted the evaluation. The calibration was validated through repeated tests, achieving a panel consistency of ≥85% both in attribute identification and intensity scoring. Under a blind assessment protocol, each attribute was independently scored by the panelists on a linear 0-10 intensity scale (0 = none, 10 = extremely intense) following established methodologies (Xue et al., 2022). Six key aroma attributes were scored: Fruity, Green, Sweet, Floral, Roasted aroma, and Overall aroma (Lu et al., 2024). Samples were prepared in accordance with the Chinese standard (GB/T 23776-2018) and subsequently filtered and transferred to a sniffing bottle.

### 2.6 Data processing and statistical analysis

Raw data preprocessing included missing value imputation and quality control (QC) sample filtering. Missing values were imputed using the k-nearest neighbors (KNN) algorithm, set to one-fifth of the minimum value for each row. Metabolites with a coefficient of variation (CV) below 0.5 in the QC samples were retained for subsequent analysis.

Multivariate statistical analyzes were performed using R software (v4.1.2). Principal Component Analysis (PCA) was conducted using the prcomp function. Orthogonal Projections to Latent Structures-Discriminant Analysis (OPLS-DA) was performed using the MetaboAnalystR package, with data subjected to log_2_ transformation and mean-centering prior to analysis (Pang et al., 2021). The established OPLS-DA models showed clear separation between groups with high model robustness (for JL1B vs. JL1S: R^2^Y = 0.99, Q^2^ = 0.984; for JL1B vs. HDB: R^2^Y = 0.999, Q^2^ = 0.971). Significantly different metabolites were identified based on a Variable Importance in Projection (VIP) score from the OPLS-DA model ≥ 1.0, an absolute log_2_ fold change (|log_2_FC|) ≥ 1.0, and a *p* < 0.05 from one-way ANOVA (Xia and Wishart, 2016).

Metabolite identification and quantification were conducted by matching retention times and mass spectra against the MWDB database. For each compound, one quantifier ion and two to three qualifier ions were monitored in SIM mode. Compound classification was primarily performed using online databases such as PubChem, Chem960, and ClassyFire. The relative content of compound i (X*_i_*, in μg/g) is calculated using the formula: X*_i_* = (V*_s_* × C*_s_*/M) × (I*_i_*/I*_s_*) × 10^−3^. In this formula, V*_s_* represents the volume of the internal standard added (in μL), C*_s_* represents the concentration of the internal standard (in μg/mL), M represents the mass of the test sample (in g), and I*_i_* and I*_s_* represent the peak areas of compound i in the test sample and the internal standard, respectively.

The relative Odor Activity Value (rOAV) was calculated to assess the contribution of each volatile compound to the overall aroma profile, using the formula: rOAV_i_ = C_i_/T_i_, where C_i_ is the relative concentration of the compound (μg/g) and T_i_ is its odor threshold (μg/g) of the compound (Xie et al., 2026). Odor thresholds and descriptors were sourced from databases including The Good Scents Company and Leffingwell & Associates. Generally, an rOAV ≥ 1 indicates a direct contribution to the sample’s aroma. Data visualization, including flavor wheel plots and heatmaps, was generated using the R packages ggplot2 and pheatmap, respectively. The flavor wheel was constructed based on the rOAV values of the aroma compounds. For pairwise comparisons among groups, one-way ANOVA was performed followed by Tukey’s Honest Significant Difference (HSD) *post hoc* test using the R aov function. All statistical analyzes were conducted using R language (v4.1.2).

## 3 Results and discussion

### 3.1 Sensory evaluation-based aroma characterization and flavor profile differentiation of black tea

**Figure 1:**
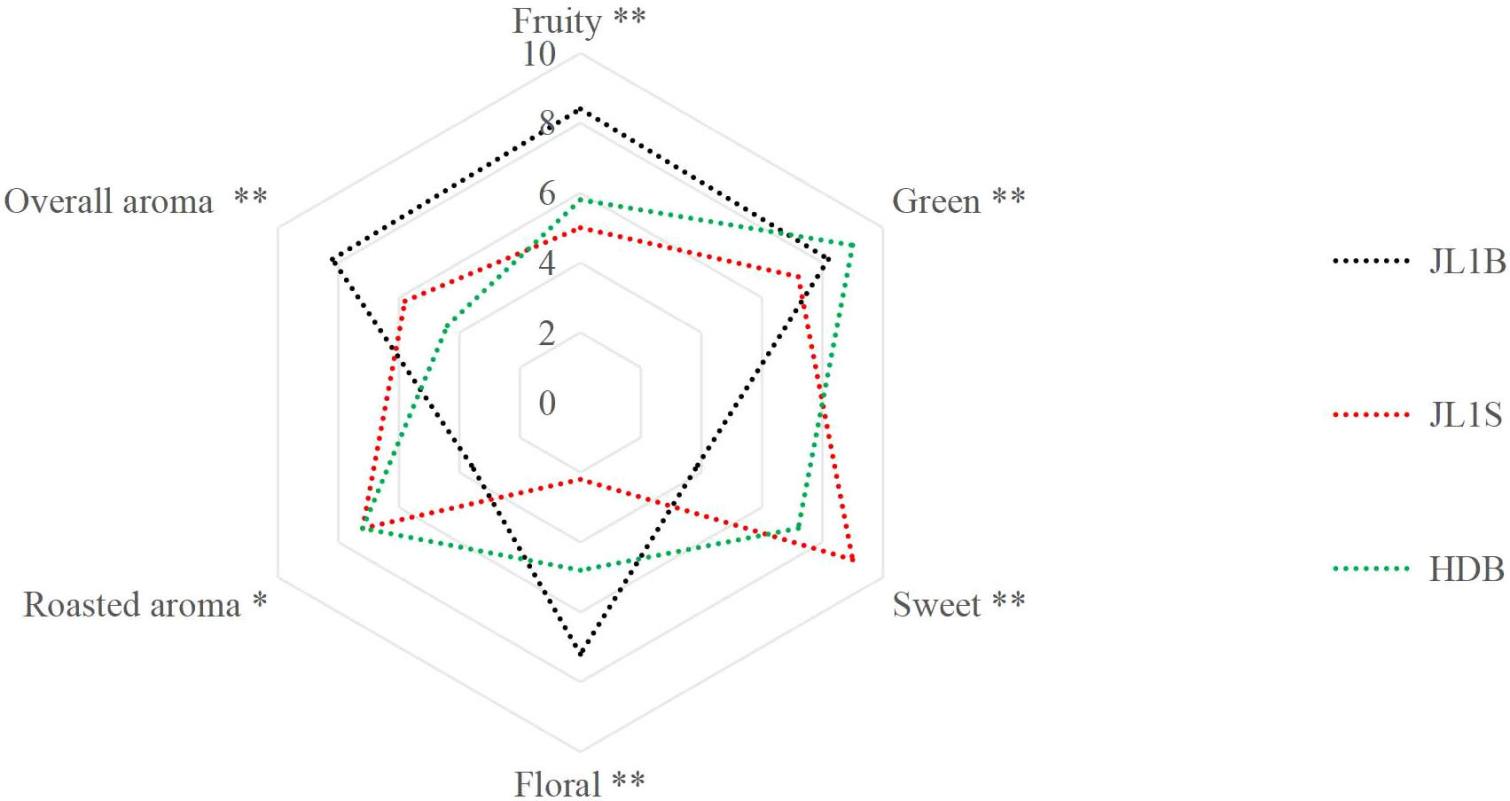
Radar map of sensory evaluation data of ‘Huangdan’ floral-fruity black tea (HDB), ‘Jinlong No.1’ floral-fruity black tea (JL1B) and ‘Jinlong No.1’ traditional black tea (JL1S). *Significant (*p* ≤ 0.05); **highly significant (*p* ≤ 0.01).

The tea cultivar was the primary determinant of the differences in volatile compounds and the ultimate aroma type, whereas the manufacturing technique acted as the key driver for the immense variation observed across different tea categories (Yuan et al., 2009). Systematic sensory evaluation revealed three distinct aroma profiles among the tea samples (**Figure 1**; **Supplementary Table 2**). JL1B presented a dominant fruity-floral profile. It exhibited highly significantly higher (*p* < 0.01) intensities in fruity (8.4 ± 0.9) compared to JL1S (5.0 ± 0.7) and HDB (5.8 ± 1.3), and in overall aroma (8.2 ± 0.8) compared to JL1S (5.8 ± 0.8) and HDB (4.4 ± 1.1). Its floral note (7.2 ± 0.8) was significantly higher (*p* < 0.05) than that of HDB (4.8 ± 0.8) and highly significantly higher (*p* < 0.01) than JL1S (2.2 ± 0.8). Conversely, it registered the lowest sweetness (3.8 ± 0.8), being highly significantly lower (*p* < 0.01) than JL1S (9.0 ± 0.6) and HDB (7.2 ± 1.3), and the lowest roasted aroma (3.6 ± 1.7), which was significantly lower (*p* < 0.05) than both JL1S (7.2 ± 1.5) and HDB (7.0 ± 2.5). The pronounced floral character of JL1B aligns with the sensory descriptor “distinct floral aroma” reported for floral-style black tea processed from ‘Huangdan’ progeny such as Huangguanyin (Yang et al., 2018).

In contrast, JL1S was characterized by a sweet-roasty profile. It showcased the highest sweetness (9.0 ± 0.6), which was highly significantly (*p* < 0.01) greater than that of JL1B and HDB. It also displayed a prominent roasted aroma (7.2 ± 1.5), which showed no significant difference with HDB but was significantly higher (*p* < 0.05) than JL1B. However, it exhibited the lowest floral note (2.2 ± 0.8), which was highly significantly lower (*p* < 0.01) than both JL1B and HDB, and the lowest fruity note (5.0 ± 0.7), being highly significantly lower (*p* < 0.01) than JL1B.

HDB demonstrated a distinct green character. Its green aroma intensity (9.0 ± 0.4) was highly significantly higher (*p* < 0.01) than both JL1S and JL1B. It also displayed moderately high sweetness (7.2 ± 1.3), which showed no significant difference from that of JL1S but was highly significantly higher (*p* < 0.01) than JL1B, and a roasted aroma (7.0 ± 2.5) that showed no significant difference from JL1S. ‘Huangdan’ black has long been described as exhibiting floral fragrance when compared with other varieties (You et al., 2025), which is broadly consistent with the distinct floral character documented in the present study.

The pronounced floral and fruity characteristics definitive of JL1B aligned with previous findings on black tea processed with shaking techniques, where mechanical disruption enhances the release of glycosidically bound volatile precursors (Yang et al., 2022). Furthermore, the distinct sensory positioning of the three samples underscores the significant combinatorial effects of genetic background and processing methodology on the final tea quality, consistent with reports on cultivar-specific aroma patterns (He et al., 2023).

### 3.2 Principal component analysis of volatile metabolites

**Figure 2:**
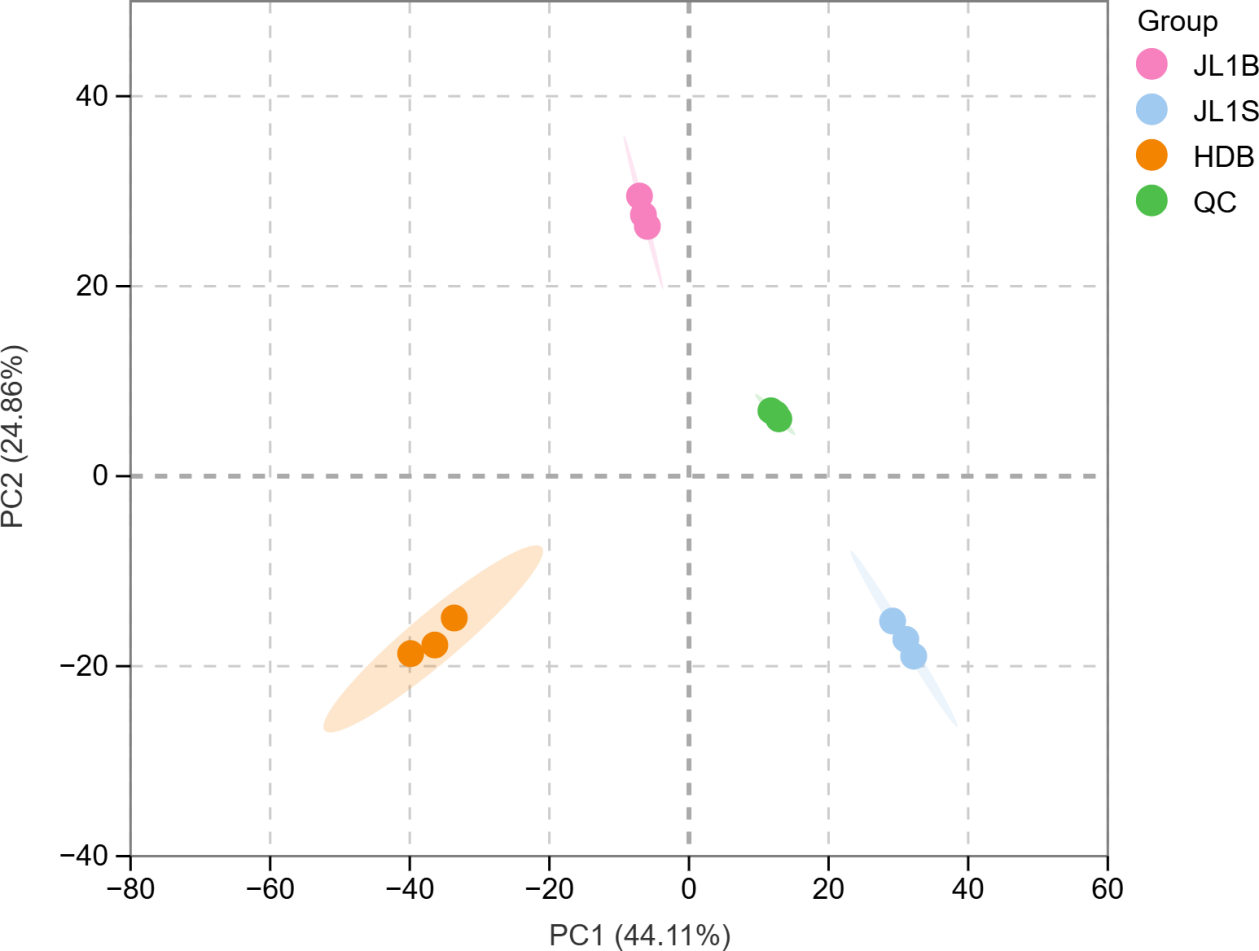
PCA score plot of volatile metabolites. PC1 and PC2 represent the first and second principal components, respectively, with the percentages indicating the proportion of the total variance explained by each component. Each point in the plot denotes an individual sample, with samples from the same group colored identically. “Group” indicates the sample categorization.

The aroma of tea is the result of the combined effects of multiple substances, depending not only on the types of aroma compounds but also on the proportion and content of different aroma compounds (Xin et al., 2019). The GC-MS volatile metabolomics method was used to identify the relative contents of volatile metabolites in the three groups of black tea. Principal component analysis (PCA) was performed on the volatile metabolites of the three sample groups. As illustrated in **Figure 2**, the contribution rate of PC1 was 44.11%, and that of PC2 was 24.86%, resulting in a cumulative contribution rate of 69.97%, which effectively captured the variance among the samples. The three groups were distributed in distinct regions in the score plot, demonstrating clear separation trends. This indicates significant differences in the volatile metabolite profiles among JL1B, JL1S, and HDB, thereby providing a metabolomic basis for their unique sensory characteristics.

### 3.3 Differential volatile metabolites between JL1B and HDB, and between JL1B and JL1S

**Figure 3:**
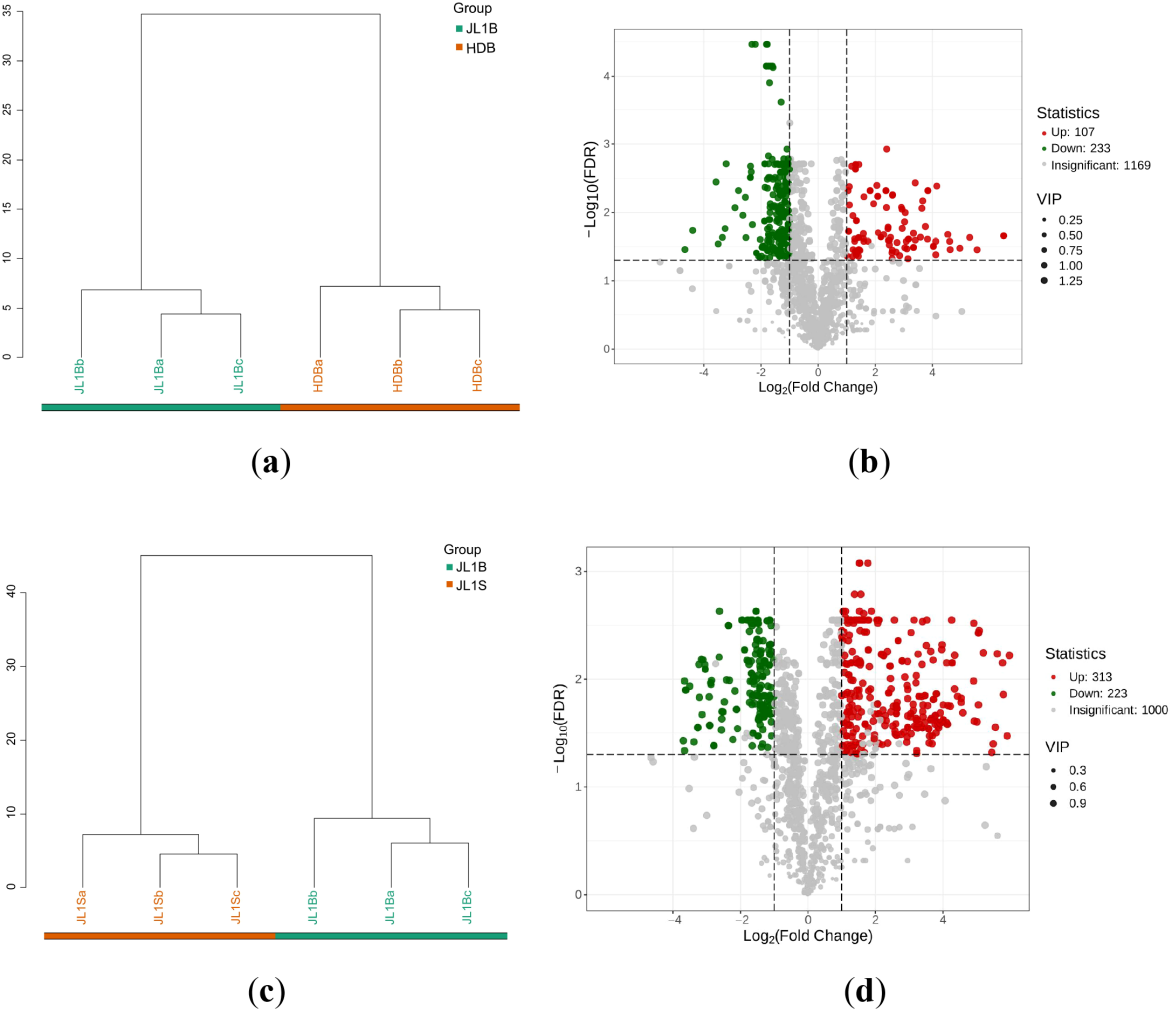
Analysis of differential metabolites between JL1B and HDB, and between JL1B and JL1S. **(a)** Hierarchical clustering dendrogram of JL1B and HDB. **(b)** Volcano plot visualizing significantly upregulated and downregulated metabolites between JL1B and HDB. **(c)** Hierarchical clustering dendrogram of JL1B and JL1S. **(d)** Volcano plot visualizing significantly upregulated and downregulated metabolites between JL1B and JL1S.

Hierarchical clustering analysis was performed to assess the similarity in metabolic profiles without prior knowledge of group labels (**Figures 3a, c**). The dendrogram revealed that samples from each group clustered together, indicating inherent group-specific metabolic patterns. Volcano plots were subsequently generated to identify differentially abundant metabolites between groups (**Figures 3b, d**).

A total of 340 significantly different volatile compounds were identified in *‘*JL1B*’* compared to *‘*HDB*’* (VIP > 1, |log_2_FC| ≥ 1.0, and *p *< 0.05), *among* which 107 were upregulated and 233 were downregulated (**Figure 3b**). As shown in **Supplementary Figure 3**, the major categories of these differential metabolites were esters (18.24%), ketones (16.47%), heterocyclic compounds (15.0%), terpenoids (12.65%), and alcohols (11.47%), which collectively represent the five most contributing classes of differential substances. The substantial metabolic divergence between these two cultivars, particularly the marked up-regulation of ester compounds in JL1B, provides a c*hemic*al basis for their distinct sensory profiles. This finding aligns with previous studies reporting that ester compounds are key contributors to the fruity aroma in premium black teas (Shi et al., 2018).

In the comparison between JL1B and JL1S derived from the same *‘*Jinlong No.1*’* line, 313 upregulated and 223 downregulated metabolites were identified in JL1B (**Figure 3d**). As shown in **Supplementary Figure 6**, terpenoids (20.34%), esters (18.28%), and heterocyclic compounds (11.94%) constituted the major categories of differential metabolites. Notably, JL1B was processed from three-to-four-leaf shoots, whereas JL1S used two-leaf raw material. The elevated ester and terpenoid levels in JL1B reflect the synergistic effect of leaf maturity and mechanical force rather than cultivar differences alone (Yin et al., 2023). This aligns with studies showing that mechanical disruption promotes the hydrolysis of glycosidic precursors and oxidative degradation of carotenoids, thereby enhancing volatile terpenoid and ester formation (Shi et al., 2018). In JL1B, the upregulation of *α*-farnesene—a sesquiterpene synthesized *de novo* via the MVA pathway—is attributed to mechanical wounding during the shaking process, which induces CsAFS gene expression (Wu et al., 2020). Mechanical damage during shaking is likely to promote hydrolysis of glycosidically bound terpenoids and upregulate terpenoid biosynthesis genes, which may thus make terpenoids the major category of differential metabolites (Gui, 2015; Wang et al., 2022). Future transcriptomic and glycoside-targeted analyzes are needed to confirm the dominant mechanism.

### 3.4 Flavor analysis of differential metabolites

**Figure 4:**
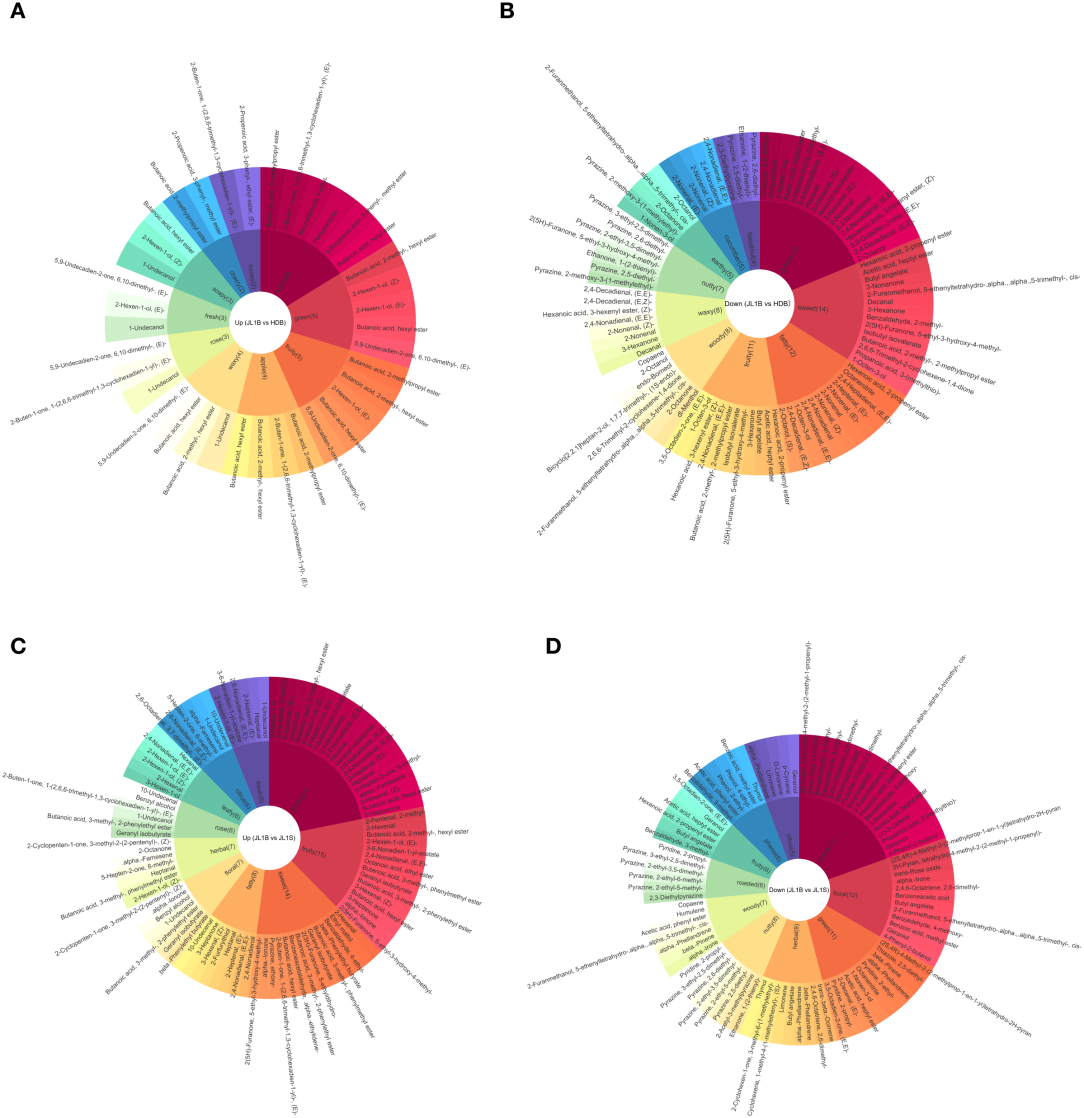
Flavor characterization of differential metabolites between JL1B and HDB, and between JL1B and JL1S. **(a)** flavor wheel based on the upregulated metabolites between JL1B and HDB. **(b)** flavor wheel based on the upregulated metabolites between JL1B and JL1S. **(c)** flavor wheel based on the downregulated metabolites between JL1B and HDB. **(d)** flavor wheel based on the downregulated metabolites between JL1B and JL1S. The innermost circle represents the aroma flavor wheel based on the upregulated metabolites. The second circle denotes the top 10 flavor characteristics with the highest number of differential metabolites annotated to each, with the numbers in parentheses indicating the count of differential metabolites associated with each flavor characteristic. The outermost circle lists the differential metabolites themselves.

Aroma components can objectively reflect the flavor characteristics of different samples and serve as a crucial indicator for evaluating flavor quality. By conducting flavor analysis of differential metabolites, the flavor traits present in the samples can be examined. In the comparison between JL1B and HDB, and between JL1B and JL1S, differential metabolites were identified based on the selection criteria (VIP > 1, |log_2_FC| ≥ 1.0, and *p* < 0.05). Additionally, the flavor characteristics annotated to these metabolites were filtered to include those with an rOAV (relative odor activity value) greater than 1. The flavor annotation diagrams for the top 10 flavor characteristics of upregulated and downregulated metabolites in different comparison groups are presented in **Figure 4**.

The flavor wheel of upregulated metabolites between JL1B and HDB is shown in **Figure 4a**. The results showed that the aroma profile of JL1B is precisely driven by a small number of upregulated metabolites with high rOAV values. These metabolites primarily contribute to sweet, fruity, and green aroma characteristics, with 6, 5, and 5 related metabolite types identified, respectively. Among them, 2-methylpropyl butanoate (Butanoic acid, 2-methylpropyl ester) contributes an apple-like scent. Within the upregulated flavor compounds, 1-undecanol (1-Undecanol), (*E*)-1-(2,6,6-trimethyl-1,3-cyclohexadien-1-yl)-2-buten-1-one, and (*E*)-5,9-undecadien-2-one, 6,10-dimethyl collectively impart rose-like floral notes. As for JL1B and JL1S (**Figure 4b**), JL1B exhibited a greater number of upregulated metabolites associated with green notes (21 compounds), followed by those contributing to fruity characteristics (15 compounds). This metabolite profile is consistent with the sensory observation that JL1B possesses pronounced fruity aromas accompanied by subtle green nuances. Key compounds responsible for the green aroma in JL1B included 3-hexen-1-ol, 2-methyl-2-pentenal, and (*E*)-2-hexenal, while the fruity character was primarily attributed to 2-methyl-2-pentenal, 2-hexenal, and 2-methylbutanoic acid hexyl ester.

In the study by Liu et al. (2021), the compound (*E*)-5,9-undecadien-2-one, 6,10-dimethyl, was detected. The research revealed that its relative content increases with the tea grade in previous studies on Dianhong Congfu black tea. Notably, Fuding black tea, which is characterized by both floral and sweet aroma attributes, had the highest relative content (1.94%), higher than that of Yibin black tea with a pure sweet aroma and Dianhong black tea with a rich sweet aroma. Indeed, in the perfumery and cosmetics sectors, (*E*)-5,9-undecadien-2-one, 6,10-dimethyl, registered as a fragrance ingredient and is permitted for use in fine fragrances up to certain concentrations (Api et al., 2021). This supports its common application in formulations where fresh, rose-like floral notes are desired.

The abundant C6 and C9 aldehydes and alcohols in JL1B are the source of its green notes. (*E*)-2-Hexenal is present only at relatively low levels in fresh tea leaves, but its content increases rapidly during the natural withering stage of black tea processing and subsequently declines during fermentation and storage (Saijo, 1977). In studies on the aroma components of black tea, (*Z*)-3-Hexen-1-ol has been frequently detected and is considered one of the key compounds contributing to the fresh and green aroma of black tea (Zhou et al., 2024). For example, Kang et al. (2019), in their research on the key aroma components of the world’s four major black teas, found that (*Z*)-3-Hexen-1-ol exhibits a high odor activity value (OAV) in black tea and makes a significant contribution to its fruity and grassy notes. Meanwhile, the peak areas representing the total abundance of (*Z*)-3-hexen-1-ol and hexanoic acid showed an increasing trend during the postharvest processing of oolong tea, which benefits from the sufficient supply of C6 aldehydes generated through the oxidation and cleavage of polyunsaturated fatty acids (PUFAs) by the LOX-HPL enzyme system (Zhou et al., 2021a).

The flavor wheel of downregulated metabolites between JL1B and HDB is shown in **Figure 4c**. In comparison with HDB, JL1B exhibited a greater number of downregulated metabolites with flavor activity. Among the major flavor compounds, those associated with green (17 compounds), sweet (14 compounds), and fatty (12 compounds) notes were predominant, while contributions to fruity and floral aromas were relatively less prominent. The decrease in compounds such as 2-propenyl hexanoate, octanenitrile, and (*E*,*E*)-2,4-heptadienal contributed to the reduction in fatty aroma. Additionally, the characteristic cucumber flavor profile was primarily defined by a group of unsaturated aldehydes, including (*E*)-2-Nonenal, 2-Nonenal, 2,4-Nonadienal, and (*E*,*E*)-2,4-Nonadienal. Notably, these key aroma compounds were also found to be shared across the fatty and green odorant categories. In comparison with JL1S (**Figure 4d**), JL1B exhibited a higher number of downregulated differential metabolites associated with sweet characteristics (17 compounds), followed by those contributing to floral (12 compounds) and green (11 compounds) notes. This metabolite profile aligned with the distinct sensory style of JL1S, which is characterized by a prominent sweet aroma accompanied by subtle floral nuances. Regarding specific compositional differences, the green attribute in JL1B was mainly derived from compounds such as 3-hexen-1-ol, 2-methyl-2-pentenal, and (*E*)-2-hexenal, while its fruity character was primarily contributed by 2-methyl-2-pentenal, 2-hexenal, and 2-methylbutanoic acid hexyl ester. In contrast, the sweet aroma of JL1S was largely attributed to compounds including 4-methyl-2-(2-methylprop-1-en-1-yl)tetrahydro-2H-pyran, trans-*β*-ocimene, and 2-methylbenzaldehyde. Meanwhile, its floral flavor was mainly contributed by (2S,4R)-4-methyl-2-(2-methylprop-1-en-1-yl)tetrahydro-2H-pyran, 4-methyl-2-(2-methylprop-1-en-1-yl)tetrahydro-2H-pyran, and trans-rose oxide.

Compared to HDB, the downregulation of C9 unsaturated aldehydes and alcohols (e.g., (*E*)-2-nonenal, 1-nonen-3-ol) in JL1B likely results from intrinsic substrate limitations between different tea lines. Previous studies have demonstrated well-marked clonal variations in fatty acid composition among tea cultivars, which directly affect the availability of precursors for LOX-derived volatiles (Ravichandran and Parthiban, 2000). However, whether this reduction is primarily driven by substrate availability, differential enzyme activity, or downstream metabolic conversion remains to be elucidated.

The prevalent down-regulation of alkylpyrazines (contributing nutty and roasted aromas) in JL1B may be indicative of a weaker Maillard reaction, which could be associated with cultivar-dependent differences in free amino acid precursors—these being the key substrates for pyrazine formation during heating (Sasaki et al., 2020; Wang et al., 2026). This could be because the more mature leaves used, combined with the shaking process, results in a Congou black tea that is more heat-tolerant, thus undergoing a less vigorous Maillard reaction compared to that in delicate black teas.

### 3.5 Characterization of relative odor activity values of commonly differential metabolites

The relative odor activity value (rOAV) is a pivotal metric for identifying key aroma compounds by relating their concentration to their sensory detection threshold (Saijo, 1977). In this study, to pinpoint the crucial metabolites responsible for the characteristic aroma of JL1B, we focused on the common differential metabolites identified from both the JL1B vs. JL1S and JL1B vs. HDB comparisons. From these, compounds with an rOAV ≥ 1 were selected as the key common metabolites for further analysis. Consequently, the rOAV analysis revealed distinct aroma profiles among the three tea samples, with compounds with an rOAV ≥ 1 considered significant contributors to the overall aroma (**Table 1**). JL1B exhibited a unique aroma pattern characterized by selective enhancement of certain fruity notes. Specifically, Butanoic acid, 2-methyl-, hexyl ester showed remarkably high rOAV values (56.9 ± 21.36) in JL1B, significantly exceeding those in JL1S (8.23 ± 1.15) and HDB (23.81 ± 2.23). This compound, known for its apple-like fruity character, appears to be a key differentiator for JL1B’s distinct fruity aroma. Similarly, Butanoic acid, hexyl ester demonstrated higher rOAV in JL1B (8.52 ± 3.09) compared to JL1S (1.87 ± 0.43) and HDB (2.93 ± 3.21), further enhancing the fruity profile. These findings align with previous studies indicating that hexyl esters of butanoic acid are key contributors to fruity aromas in fermented foods and beverages, including tea. For instance, Rawat et al. (2007) highlighted that these esters are formed during the fermentation process via esterification of free fatty acids and alcohols, and their accumulation is influenced by microbial activity and processing conditions. The elevated levels in JL1B suggest that its processing may favor ester formation, possibly due to specific microbial consortia or fermentation parameters.

**Table 1:** Comparative relative odor activity values (rOAV) of key differential aroma compounds among JL1B, JL1S and HDB.

Compounds	Quantitative ion	Qualitative ion	NIST RI	Calculated RI	CAS	Threshold (mg/kg)	rOAV		
							JL1S	JL1B	HDB
2-Furanmethanol, 5-ethenyltetrahydro-*α*,*α*,5-trimethyl-, cis-	59	94	1074	1072.00	5989-33-3	0.32	22.54 ± 0.39 aA	9.9 ± 1.73 bB	21.71 ± 1.82 aA
Butanoic acid, hexyl ester	71	89	1190.35	1189.83	2639-63-6	0.203	1.87 ± 0.43 bB	8.52 ± 3.09 aA	2.93 ± 3.21 bB
Cyclohexene, 1-methyl-4-(1-methylethylidene)-	93	121	1090.66	1088.20	586-62-9	0.2	28.52 ± 0.28 bA	10.61 ± 1.97 cB	35.24 ± 2.22 aA
5-Hepten-2-one, 6-methyl-	108	58	987.69	986.01	110-93-0	0.05	4.53 ± 0.68 bB	11.76 ± 4.19 bB	62.73 ± 4.27 aA
Butanoic acid, 2-methyl-, hexyl ester	103	57	1236	1235.60	10032-15-2	0.022	8.23 ± 1.15 cC	56.9 ± 21.36 aA	23.81 ± 2.23 bB
1,3-Dithiane, 2-methyl-	119	74	1034	1042.82	6007-26-7	0.02	2.12 ± 0.05 cB	6.66 ± 3.05 aA	3.04 ± 3.04 bB
3-Nonanone	85	113	1090	1088.13	925-78-0	0.017	67.06 ± 0.48 bA	27.64 ± 4.31 cB	85.13 ± 5.05 aA
Butyl angelate	83	100	1091	1088.17	7785-64-0	0.013	178 ± 1.48 bA	67.49 ± 12.21 cB	232.34 ± 14.14 aA
Benzaldehyde, 2-methyl-	91	119	1064	1059.22	529-20-4	0.0096	59.88 ± 0.75 aA	18.82 ± 8.18 cC	52.85 ± 8.6 bB
Pyrazine, 3-ethyl-2,5-dimethyl-	135	136	1081	1088.36	13360-65-1	0.0086	3.49 ± 0.23 aA	1.25 ± 0.46 bB	3.86 ± 0.37 aA
Pyrazine, 2,5-diethyl-	107	135	1091	1088.24	13238-84-1	0.007	23.72 ± 0.29 aA	9.28 ± 1.83 bB	25.27 ± 2.03 aA
1,2-Dithiane	39	60	1087	1087.92	505-20-4	0.002	28.2 ± 0.45 aAB	13.34 ± 0.75 bB	34.49 ± 0.68 aA
Ethanone, 1-(2-thienyl)-	111	126	1092	1088.24	88-15-3	0.001	5251.78 ± 58.56 bB	1946.03 ± 382.97 cC	6416.17 ± 423.3 aA
1-Nonen-3-ol	72	43	1080	1088.10	21964-44-3	0.001	812.71 ± 7.25 bB	295.1 ± 56.44 cC	1066.79 ± 62.54 aA
Non-8-enal	94	67	1094	1088.20	39770-04-2	0.0002	41330.3 ± 391.05 bA	15524.66 ± 2909.96 cB	53263.47 ± 3213.01 aA
Octanenitrile	68	96	1081	1088.06	124-12-9	0.00013	32790.57 ± 547.91 bB	11951.4 ± 2132.92 cC	43028.13 ± 2312.21 aA
1-Nonen-3-one	55	70	1076	1072.00	24415-26-7	0.000001	2291072.4 ± 31222.39 aA	1012515.75 ± 182902.4 bB	2212283.97 ± 192978.58 aA

Data are shown as mean ± standard deviation (SD) of three biological replicates (*n* = 3). All data are rounded to two decimal places. Different lowercase letters within the same row indicate statistically significant differences among the tea sample groups (JL1B, JL1S, HDB) as determined by one-way ANOVA with Tukey’s *post hoc* test (*p* < 0.05). Different lowercase letters indicate significant difference (*p* < 0.05), and different uppercase letters indicate extremely significant difference (*p* < 0.01) among treatments.

Odor thresholds and descriptors were sourced from databases including The Good Scents Company and Leffingwell & Associates.

The green and fatty aroma characteristics showed a gradient pattern across samples. Compounds including 1-Nonen-3-ol, 1-Nonen-3-one, Non-8-enal, and Octanenitrile exhibited the highest rOAV values in HDB, intermediate in JL1S, and lowest in JL1B. This gradient suggests that JL1B possesses milder green and fatty notes compared to the other two samples. For instance, 1-Nonen-3-one, despite its extremely low threshold (0.000001 mg/kg), showed significantly lower rOAV in JL1B (1012515.75) than in both JL1S (2291072.40) and HDB (2212283.97).

These C9 unsaturated alcohols, ketones, and aldehydes are well-documented degradation products of linoleic and linolenic acids via lipoxygenase (LOX) pathways, especially during the early stages of tea processing such as withering and rolling (Yang et al., 2013). Their lower rOAV in JL1B suggests a suppression of LOX activity or enhanced degradation/conversion during processing, possibly due to differences in enzyme activity or oxidation conditions.

Pyrazine derivatives, known for their nutty and roasted aromas, showed distinct distribution patterns. Both 3-ethyl-2,5-dimethyl-pyrazine and 2,5-diethyl-pyrazine maintained higher rOAV values in JL1S and HDB compared to JL1B, indicating JL1B’s reduced roasted character. Pyrazines are Maillard reaction products typically formed during thermal processing such as roasting or baking. Their lower abundance in JL1B implies that this variety may undergo milder or shorter heat treatment, or possess lower precursor levels (e.g., amino acids and reducing sugars), as also noted by Chen et al. (2010) in oolong tea processing. This could be intentional to preserve more volatile fruity esters, which are heat-sensitive, thus explaining the dominance of fruity over roasted notes in JL1B.

Notably, several compounds displayed similar trends between JL1S and HDB, with both showing higher rOAV values than JL1B. These include 5-ethenyltetrahydro-*α*,*α*,5-trimethyl-cis-2-furanmethanol, Butyl angelate, 2-methyl-benzaldehyde, and 1,2-Dithiane, suggesting that JL1B lacks certain sweet and herbal notes present in the other two varieties. Among these, 2-methyl-benzaldehyde has been reported to contribute sweet, almond-like aromas in tea (Kumazawa and Masuda, 2002; Schuh and Schieberle, 2006). Meanwhile, 1,2-dithiane is a sulfur-containing compound associated with herbal and slightly garlicky notes (Kang et al., 2019). This type of volatile is likely derived from the degradation of sulfur-containing amino acids (e.g., cysteine, methionine) during fermentation or heat processing, as reviewed in the general pathway of tea aroma formation (Ho et al., 2015). The lower rOAV of these compounds in JL1B suggests a different metabolic pathway or reduced precursor availability, possibly due to varietal-specific enzyme expression or microbial community differences during fermentation.

### 3.6 Flavor network construction and traceability of key aroma compounds

**Figure 5:**
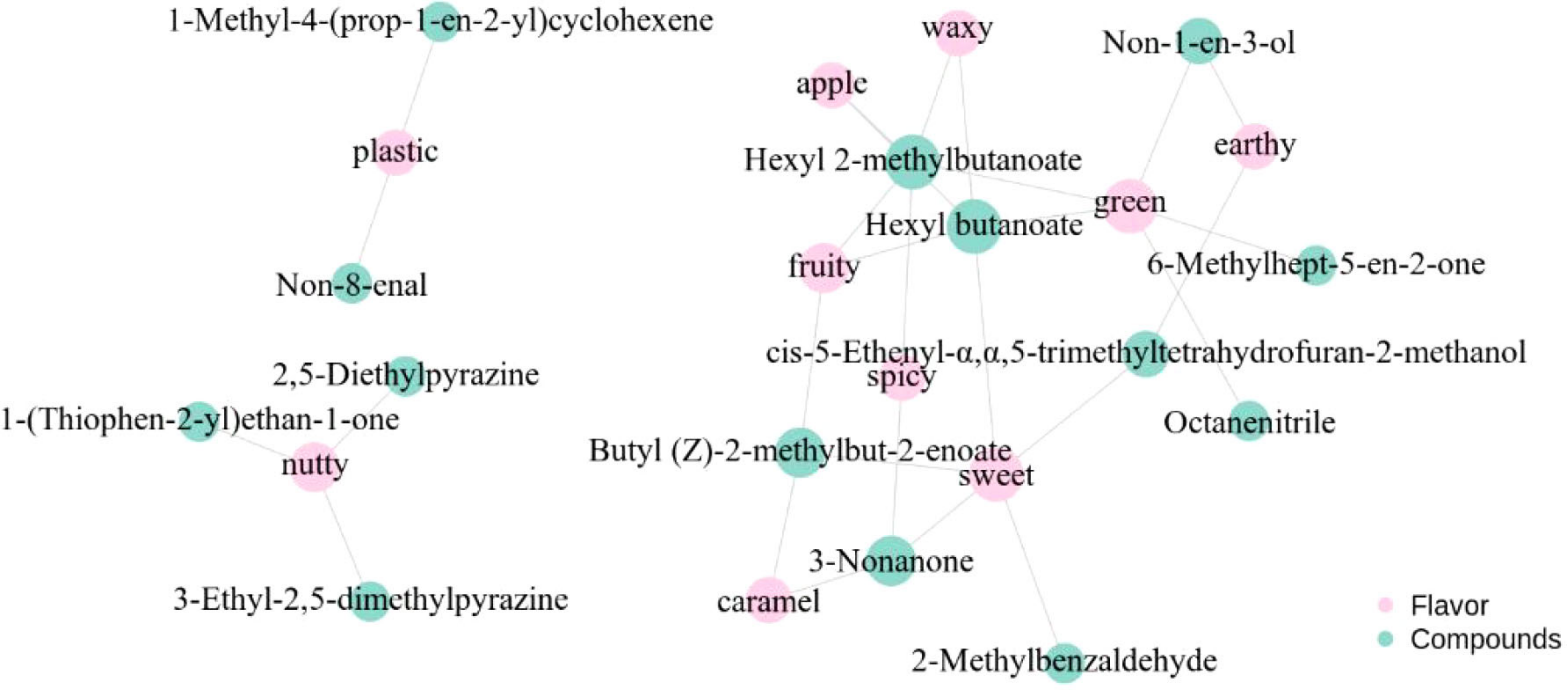
Flavor network of key aroma compounds using the top 10 differential aroma-active metabolites.

A flavor network was constructed from the 10 most differential aroma-active metabolites, which collectively spanned green, fruity, sweet, nutty, apple-like notes, etc. (**Figure 5**). Fruity perceptions, including apple-like notes, were chiefly anchored by butyl (*Z*)-2-methylbut-2-enoate, hexyl butanoate and hexyl 2-methylbutanoate. This aligns with Kumazawa and Masuda (2002), who demonstrated that these esters achieve the highest rOAV weights within the fruit cluster when their concentrations exceed odor thresholds by 8- to 14-fold, as observed in Dianhong black tea samples. Hexyl 2-methylbutanoate acts as a dual node, linking general fruitiness to the characteristic apple note. This is consistent with Kang et al. (2019), who reported this compound as the top-ranking rOAV contributor among esters in black tea, suggesting its pivotal role in bridging diverse fruity nuances. The significant increase in esters—particularly hexyl butanoate and hexyl 2-methylbutanoate—in the floral-style teas (JL1B and HDB) is mechanistically attributed to an enzymatic cascade triggered by the shaking treatment. Mechanical stress damages leaf tissue, rapidly up-regulating lipoxygenase (LOX), alcohol dehydrogenase (ADH) and alcohol acyltransferases (AATs) (Zhou et al., 2021b). LOX first oxidizes linolenic acid to volatile aldehydes; ADH then reduces these to corresponding alcohols; finally, AATs esterify the alcohols with activated acyl-CoA to form the characteristic fruity esters (Zhou et al., 2021c). Xue et al. (2022) consider that the elevated levels of these apple-like, low-threshold esters are key contributors to the distinct fruity aroma of the shaken teas. Ho et al. (2015) maintain that the perception of sweet dimensions in black tea is often a result of compositional synergy, a well-established principle in tea flavor chemistry whereby the overall sensory impression arises from the combined interaction of multiple volatiles rather than individual compounds. In a specific case, Schuh and Schieberle (2006) identified 2-methylbenzaldehyde as a key odorant contributing almond-like sweetness in Darjeeling black tea, and this compound can cooperatively frame the sweet note with 3-nonanone. Meanwhile, Ho et al. (2015) also suggest that esters like hexyl butanoate often function as bridges connecting sweet and fruity aromatic modules, further illustrating this complex synergistic network that underlies holistic flavor perception, which is consistent with established principles of tea flavor chemistry.

### 3.7 Correlation analysis between key differential volatile metabolites and sensory attributes

**Figure 6:**
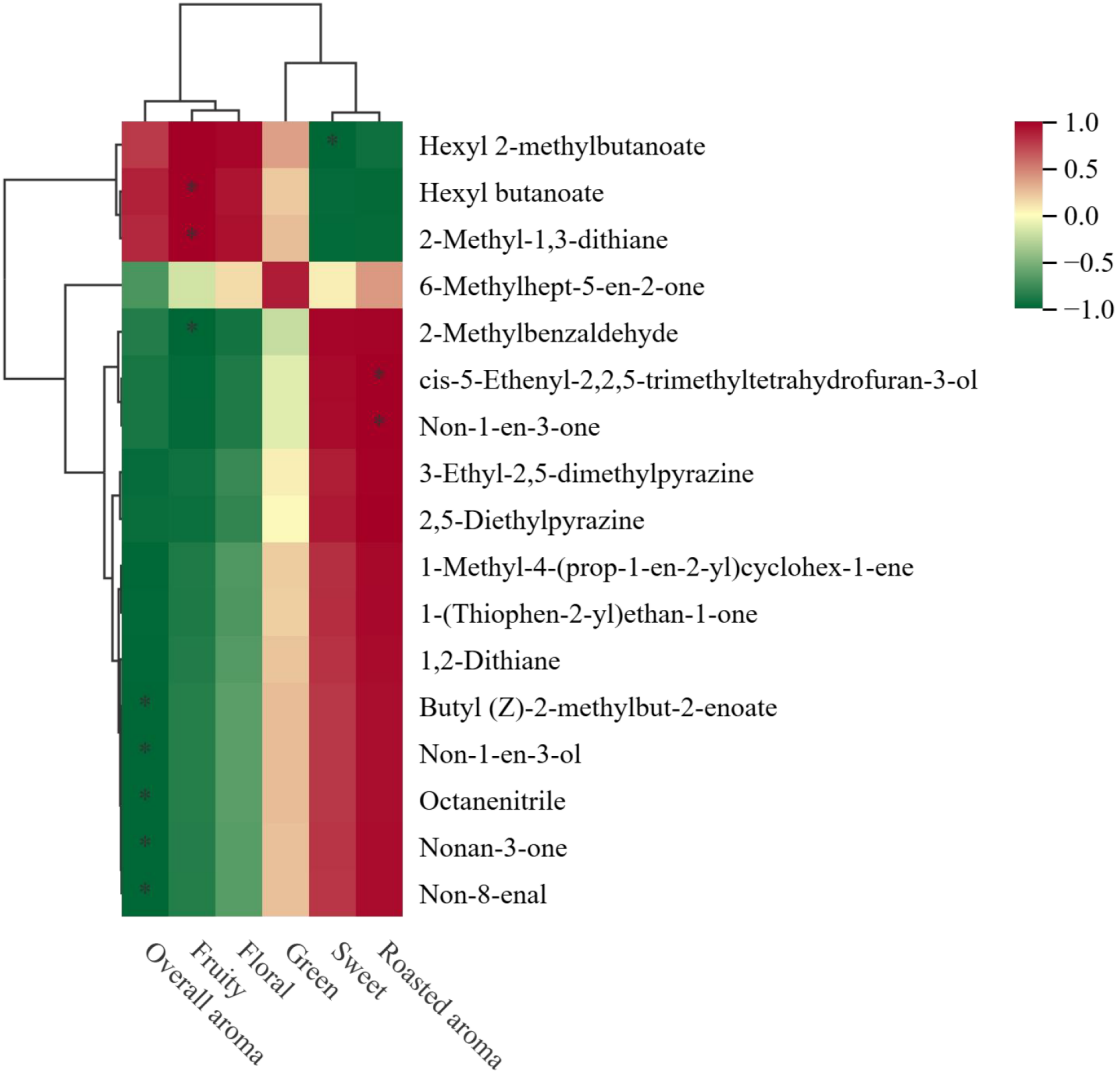
Correlation heatmap between key volatile compounds and sensory attributes in black tea samples. The analysis is based on Pearson correlation coefficients. * indicates significant correlations at *p* < 0.05.

Correlation analysis revealed significant relationships between key volatile compounds and the sensory attributes of the tea samples (**Figure 6**). Hexyl 2-methylbutanoate and hexyl butanoate both demonstrated significantly positive correlations with fruity aroma (*p* < 0.05). This aligns with their well-established odor profiles. Dunkel et al. (2014) characterize hexyl butanoate by sweet, fruity, and pineapple-like aromas and identify it as a key volatile in apples and other fruits. Zhu et al. (2015) note that hexyl 2-methylbutanoate often imparts complex, apple-like fruity nuances. Their marked correlation with fruity aroma substantiates their direct role in defining the fruity character of the evaluated teas.

Notably, hexyl 2-methylbutanoate exhibited a significant negative correlation with sweetness (*p* < 0.05). Feng et al. (2019) suggest that while this ester enhances fruity notes, its distinct aroma profile, which can carry green or fermented undertones, might antagonize the perception of sweetness within the tea matrix.

Conversely, non-1-en-3-ol, non-1-en-3-one, non-8-enal, and octanenitrile were significantly negatively correlated with overall aroma intensity (*p* < 0.05). These compounds are typical oxidation products of fatty acids via the lipoxygenase pathway and are commonly associated with green, fatty, and earthy odors. Guo et al. (2021) note that non-1-en-3-one, known for its extremely low odor threshold, possesses a mushroom-like, earthy scent that can be undesirable at higher concentrations. The negative impact of these compounds indicates that green-fatty notes are likely detrimental to the overall aroma quality of the floral-style black teas under investigation.

A noteworthy finding was the significantly positive correlation between 2-methyl-1,3-dithiane and fruity aroma (*p* < 0.05). This is intriguing because sulfur-containing compounds are frequently linked to savory, roasted, or occasionally unpleasant notes in foods. Its positive association with fruity aroma implies a potential synergistic effect, whereby it may enhance the complexity of fruity notes within the tea, rather than contributing a direct fruity scent itself.

The observed correlation patterns underscore that the influence of a volatile compound on the overall sensory profile is not determined solely by its aroma characteristics, but also by its interactions with other constituents within the food matrix. This complexity highlights the necessity of integrating chemical correlation data with an understanding of inherent aroma properties in the interpretation of sensory outcomes, which is consistent with established principles of tea flavor chemistry.

## 4 Conclusions

This study systematically investigated the aroma differentiation between the novel tea cultivar ‘Jinlong No.1’ and its ancestral cultivar ‘Huangdan’, as well as the impact of different processing types on the aroma profile of the new cultivar, by integrating GC-MS/MS-based volatile metabolomics, quantitative rOAV evaluation, and sensory analysis. Results showed that the floral-fruity black tea (JL1B) produced from ‘Jinlong No.1’ achieved the highest sensory scores for overall aroma (8.2 ± 0.8), floral (7.2 ± 0.8), and fruity (8.4 ± 0.9) attributes. Metabolomic profiling identified 17 common differential volatile metabolites, including key esters such as hexyl 2-methylbutanoate. Compared with previous studies, this work not only established a multi-dimensional analytical framework to systematically unravel the cultivar-processing interaction but also integrated metabolomic and sensory data to identify key aroma-active compounds and their metabolic basis. It should be noted that this study’s findings are based on tea samples produced in a single location and season. Future research incorporating multi-year and multi-location trials is recommended to confirm the robustness and generalizability of these aroma markers. Furthermore, the current research primarily relied on correlation-based metabolomic analysis, leaving the enzymatic mechanisms and regulatory networks underlying the formation of these differential metabolites largely unexplored. Future studies will employ transcriptomic approaches to validate expression patterns of key genes in relevant biosynthetic pathways, with the aim of elucidating the molecular-level biosynthetic mechanisms of these aroma compounds and providing a more comprehensive theoretical foundation for precise flavor quality regulation in black tea.

## Informed consent statement

Informed consent was obtained from all subjects involved in the study.

## Data Availability

The datasets presented in this study can be found in online repositories. The names of the repository/repositories and accession number(s) can be found in the article/**Supplementary Material**.

## References

[B1] Api A. M. Belsito D. Botelho D. Bruze M. Burton G. A. Buschmann J. . (2021). RIFM fragrance ingredient safety assessment, 6,10-dimethylundeca-5,9-dien-2-one, CAS registry number 689-67-8. Food Chem. Toxicol. 149, 111981. doi: 10.1016/j.fct.2021.112489. PMID: 34390813

[B2] Chen Y. Jiang Y. Duan J. Shi J. Xue S. Kakuda Y. (2010). Variation in catechin contents in relation to quality of 'Huang Zhi Xiang' Oolong tea (Camellia sinensis) at various processing stages. Food Chem. 119, 648–652. doi: 10.1016/j.foodchem.2009.07.014. PMID: 41936479

[B3] Dunkel A. Steinhaus M. Kotthoff M. Nowak B. Krautwurst D. Schieberle P. (2014). Nature's chemical signatures in human olfaction: a foodborne perspective for future biotechnology. Angew. Chem. Int. Ed. 53, 7124–7143. doi: 10.1002/anie.201309508. PMID: 24939725

[B4] Feng Z. Li M. Li Y. Wang Y. Zhang L. Wan X. . (2019). Tea aroma formation from six model manufacturing processes. Food Chem. 285, 347–354. doi: 10.1016/j.foodchem.2019.01.174. PMID: 30797356

[B5] Gong L. J. Bo J. H. Du Z. R. Li J. Sun H. Y. Chen Y. Q. . (2021). Metabolomic analysis of metabolite changes during the fermentation of Congou black tea based on widely targeted metabolomics. Sci. Technol. Food. Industry 42, 8–16. doi: 10.13386/j.issn1002-0306.2021030361

[B6] Gui C. D. (2015). Does enzymatic hydrolysis of glycosidically bound volatile compounds really contribute to the formation of volatile compounds during the oolong tea manufacturing process? J. Agric. Food. Chem. 63, 6905–6914. doi: 10.1021/acs.jafc.5b02741. PMID: 26212085

[B7] Guo X. Ho C. T. Schwab W. Wan X. (2021). Effect of the roasting degree on flavor quality of large-leaf black tea. Food Chem. 347, 129016. doi: 10.1016/j.foodchem.2021.129016. PMID: 33486364

[B8] He C. Zhou J. Li Y. Zhang D. Ntezimana B. Zhu J. . (2023). The aroma characteristics of oolong tea are jointly determined by processing mode and tea cultivars. Food. Chemistry: X 18, 100730. doi: 10.1016/j.fochx.2023.100730. PMID: 37397208 PMC10314214

[B9] Ho C. T. Zheng X. Li S. (2015). Tea aroma formation. Food Sci. Hum. Wellness 4, 9–27. doi: 10.1016/j.fshw.2015.04.001. PMID: 41936479

[B10] Kang S. Yan H. Zhu Y. Liu X. Lv H. P. Zhang Y. . (2019). Identification and quantification of key odorants in the world's four most famous black teas. Food Res. Int. 121, 73–83. doi: 10.1016/j.foodres.2019.03.009. PMID: 31108802

[B11] Kumazawa K. Masuda H. (2002). Identification of potent odorants in different green tea varieties using flavor dilution technique. J. Agric. Food. Chem. 50, 5660–5663. doi: 10.1021/jf020498j. PMID: 12236694

[B12] Liu X. Y. Li G. Lv C. Y. Xiong C. Y. Peng Y. (2021). SPME/GC-MS analysis of aroma quality of black tea from different producing areas. Sci. Technol. Food. Industry 42, 228–235. doi: 10.13386/j.issn1002-0306.2020060025

[B13] Lu M. Sheng C. Ke H. Li T. Liu Q. Zhang J. . (2024). Revealing the differences in aroma of black tea under different drying methods based on GC–MS, GC-O. Food. Chemistry: X 23, 101782. doi: 10.1016/j.fochx.2024.101782. PMID: 39280227 PMC11402106

[B14] Luo L. F. Liang G. Z. Mo X. Y. Feng H. Y. Liu H. Y. Li Z. P. . (2017). Preliminary study on the processing technology of floral black tea 'Huang Guanyin'. China Hortic. Abstracts 1, 219–220. doi: 10.3969/j.issn.1672-0873.2017.01.085. PMID: 35900448

[B15] Pang Z. Chong J. Zhou G. de Lima Morais D. A. Chang L. Barrette M. . (2021). MetaboAnalyst 5.0: Narrowing the gap between raw spectra and functional insights. Nucleic Acids Res. 49, W388–W396. doi: 10.1093/nar/gkab382. PMID: 34019663 PMC8265181

[B16] Paonessa R. Nardi M. Di Gioia M. L. Olivito F. Oliverio M. Procopio A. (2018). Eco-friendly synthesis of lipophilic EGCG derivatives and antitumor and antioxidant evaluation. Nat. Prod. Commun. 13, 1934578X1801300905. doi: 10.1177/1934578X1801300905. PMID: 41930703

[B17] Ravichandran R. Parthiban R. (2000). Lipid occurrence, distribution and degradation to flavour volatiles during tea processing. Food Chem. 68, 7–13. doi: 10.1016/S0308-8146(99)00143-0

[B18] Rawat R. Gulati A. Babu G. D. K. Acharya R. (2007). Characterization of volatile components of Kangra orthodox black tea by gas chromatography-mass spectrometry. Food Chem. 105, 229–235. doi: 10.1016/j.foodchem.2006.11.014. PMID: 41936479

[B19] Saijo R. (1977). Mechanism of developing black tea aroma with special reference to alcoholic compounds. Japan Agric. Res. Q. 11, 216–220. Available online at: https://www.jircas.go.jp/sites/default/files/publication/jarq/11-4-216-220_0.pdf.

[B20] Sasaki T. Yuikawa N. Tanihiro N. Michihata T. Enomoto T. (2020). The effects of roasting conditions on the physical appearance traits and aroma and taste components of roasted stem tea. Food Sci. Technol. Res. 26, 643–654. doi: 10.3136/fstr.26.643. PMID: 37595691

[B21] Schuh C. Schieberle P. (2006). Characterization of the key aroma compounds in the beverage prepared from Darjeeling black tea: quantitative differences between tea leaves and infusion. J. Agric. Food. Chem. 54, 916–924. doi: 10.1021/jf052495n. PMID: 16448203

[B22] Shan Z. G. Zhang C. H. Shan L. J. Wang R. F. (2022). Effect of withering methods on the chemical components of Yunnan black tea. Trop. Agric. Eng. 46, 21–23.

[B23] Shi Y. Di T. Yang S. Wu L. Chen Y. Xia T. . (2018). Changes in aroma components in the processing of flowery black tea. Food. Sci. 39, 167–175. doi: 10.7506/spkx1002-6630-201808027

[B24] Wang H. Q. Xiao M. X. An Q. Zheng F. L. Xiao M. J. Dai Q. Y. (2022). Effect of sunlight and mechanical damage on aroma quality of postharvest fresh tea leaves. J. Fujian Agric. Forestry Univ. (Natural Sci. Edition) 51, 162–170. doi: 10.13323/j.cnki.j.fafu(nat.sci.).2022.02.003

[B25] Wang Y. P. Luo Q. Q. Xu P. F. Zhu W. F. Xie X. Y. Wu J. H. . (2026). Quality analysis of autumn black tea made from different tea cultivars in southern Jiangxi. Chin. Tea 48, 64–69. Available online at: https://link.cnki.net/urlid/33.1117.S.20260209.1330.004.

[B26] Wu Q. Y. Zhou Z. W. Wu Q. Y. Ni Z. X. Lai Z. X. Sun Y. (2020). Screening and expression analysis of key regulator gene associated with α-farnesene formation during manufacturing process of oolong tea. Sci. Technol. Food. Industry 41, 135–142. doi: 10.13386/j.issn1002-0306.2020.15.022

[B27] Xia J. Wishart D. S. (2016). Using MetaboAnalyst 3.0 for comprehensive metabolomics data analysis. Curr. Protoc. Bioinf. 55, 14.10.1–14.10.91. doi: 10.1002/cpbi.11. PMID: 27603023

[B28] Xie F. Zhang H. Jiang S. Xiao M. Hu H. Song Y. . (2026). Unraveling the impacts of different processing methods on hawk tea's volatiles and flavor via non-targeted volatilomics approach. Food. Chemistry: X 33, 103520. doi: 10.1016/j.fochx.2026.103520. PMID: 41623970 PMC12853061

[B29] Xin D. D. Zhang H. Li H. B. Mo H. Z. (2019). Research progress on main aroma components in volatile components of different tea types. J. Henan Institute Sci. Technol. (Natural Sci. Edition) 47, 21–28. doi: 10.3969/j.issn.1008-7516.2019.06.005. PMID: 35900448

[B30] Xu M. Gu M. Chen J. Wei M. Chen Q. Wu W. . (2024). Flavor quality analysis of high-aroma black tea of Jinmudan and Jinguanyin based on metabolomics. Food. Sci. 45, 150–161. doi: 10.7506/spkx1002-6630-20231108-095

[B31] Xue J. Liu P. Yin J. Wang W. Zhang J. Wang W. . (2022). Dynamic changes in volatile compounds of shaken black tea during its manufacture by GC × GC-TOFMS and multivariate data analysis. Foods 11, 1228. doi: 10.3390/foods11091228. PMID: 35563951 PMC9102106

[B32] Yan Z. Zhong Y. Duan Y. Chen Q. Li F. (2020). Antioxidant mechanism of tea polyphenols and its impact on health benefits. Anim. Nutr. 6, 115–123. doi: 10.1016/j.aninu.2020.01.001. PMID: 32542190 PMC7283370

[B33] Yang J. Y. Feng H. Y. He W. Xu D. Y. Liang G. Z. Luo L. F. . (2018). Processing technology and content analysis of floral aroma type Huangguanyin black tea. J. Anhui Agric. Sci. 46, 155–157. doi: 10.13989/j.cnki.0517-6611.2018.34.048

[B34] Yang J. H. Zhou H. C. Liu Y. Q. Wang H. Huang J. Q. Lei P. D. (2022). Differences in aroma components of black tea processed from different tea cultivars in Huangshan by using headspace-solid phase microextraction-gas chromatography-mass spectrometry and odor activity value. Food. Sci. 43, 235–241. doi: 10.7506/spkx1002-6630-20210726-301

[B35] Yang Z. Baldermann S. Watanabe N. (2013). Recent studies of the volatile compounds in tea. Food Res. Int. 53, 585–599. doi: 10.1016/j.foodres.2013.02.011. PMID: 41936479

[B36] Ye F. Qiao X. Gui A. Wang S. Liu P. Wang X. . (2021). Metabolomics provides a novel interpretation of the changes in main compounds during black tea processing through different drying methods. Molecules 26, 6739. doi: 10.3390/molecules26216739. PMID: 34771147 PMC8587435

[B37] Yin X. Xiao Y. Wang K. Wu W. Huang J. Liu S. . (2023). Effect of shaking manner on aroma quality of Hunan black tea and identification of key floral aroma-active compounds. Food Res. Int. 174, 113515. doi: 10.1016/j.foodres.2023.113515. PMID: 37986507

[B38] You X. M. Li X. L. Chen Z. H. Kong X. R. Shan R. Y. Chen C. S. (2025). Suitability identification and aroma characteristics of 'Yilu Xiang' and 'Yelaixiang' tea strains for multiple tea categories. J. Northwest. A&F Univ. (Natural Sci. Edition) 11, 1–12. doi: 10.13207/j.cnki.jnwafu.2025.11.017

[B39] Yuan H. Cao G. Hou X. Huang M. Du P. Tan T. . (2022). Development of a widely targeted volatilomics method for profiling volatilomes in plants. Mol. Plant 15, 189–202. doi: 10.1016/j.molp.2021.09.003. PMID: 34509640

[B40] Yuan H. Jiangfang Y. Liu Z. Su R. Li Q. Fang C. . (2024). WTV2.0: A high-coverage plant volatilomics method with a comprehensive selective ion monitoring acquisition mode. Mol. Plant 17, 972–985. doi: 10.1016/j.molp.2024.04.012. PMID: 38685707

[B41] Yuan H. B. Yin J. F. Ye G. Z. Xu Y. Wang F. (2009). Research progress on tea aroma types and characteristic substances (continued). China Tea 31, 11–13. doi: 10.3969/j.issn.1000-3150.2009.09.004. PMID: 35900448

[B42] Zhang Y. Chen J. Xu J. Yang X. Wang S. Zeng Y. . (2025). Effects of tea polyphenols and tea polysaccharides on improving nonylphenol-induced depression-like behavior, monoamine neurotransmitter disorder, and neuronal pyroptosis. Ecotoxicology Environ. Saf. 295, 118166. doi: 10.1016/j.ecoenv.2025.118166. PMID: 40209346

[B43] Zhou W. Kong W. Yang C. Feng R. Xi W. (2021c). Alcohol acyltransferase is involved in the biosynthesis of C6 esters in apricot (Prunus Armeniaca L.) fruit. Front. Plant Sci. 12. doi: 10.3389/fpls.2021.763139. PMID: 34868159 PMC8636060

[B44] Zhou Q. Liang D. Ling C. Gao L. Ling Z. (2024). Characterization of the volatile components in the processing of Citri Exocarpium Rubrum black tea based on HS-SPME and GC/MS. Food. Sci. Nutr. 12, 7913–7923. doi: 10.1002/fsn3.4374. PMID: 39479602 PMC11521720

[B45] Zhou Z. Liu B. Wu Q. Bi W. Ni Z. Lai Z. . (2021b). Formation and regulation of aroma-related volatiles during the manufacturing process of Wuyi Rougui tea via LOX-HPL pathway. J. Food. Sci. Biotechnol. 40, 100–111. doi: 10.3969/j.issn.1673-1689.2021.01.013. PMID: 35900448

[B46] Zhou Z. W. Wu Q. Y. Ni Z. X. Hu Q. C. Yang Y. Zheng Y. C. . (2021a). Metabolic flow of C6 volatile compounds from LOX-HPL pathway based on airflow during the post-harvest process of oolong tea. Front. Plant Sci. 12. doi: 10.3389/fpls.2021.738445. PMID: 34745173 PMC8569582

[B47] Zhu J. Chen F. Wang L. Niu Y. Yu D. Shu C. . (2015). Characterization of the key aroma volatile compounds in cranberry (Vaccinium macrocarpon Ait.) using gas chromatography-olfactometry (GC-O) and odor activity value (OAV). J. Agric. Food. Chem. 63, 110–118. doi: 10.1021/acs.jafc.6b01150. PMID: 27265519

