# Supplementary figures and images for: Volatile metabolomics combined with rOAV and sensory evaluation reveals the aroma basis of black tea processed from the novel tea line ‘Jinlong No.1’ and its progenitor ‘Huangdan’
Source: Front Plant Sci. 2026 Apr 22;17:1803743. doi: 10.3389/fpls.2026.1803743 (PMC13143945; doi:10.3389/fpls.2026.1803743)

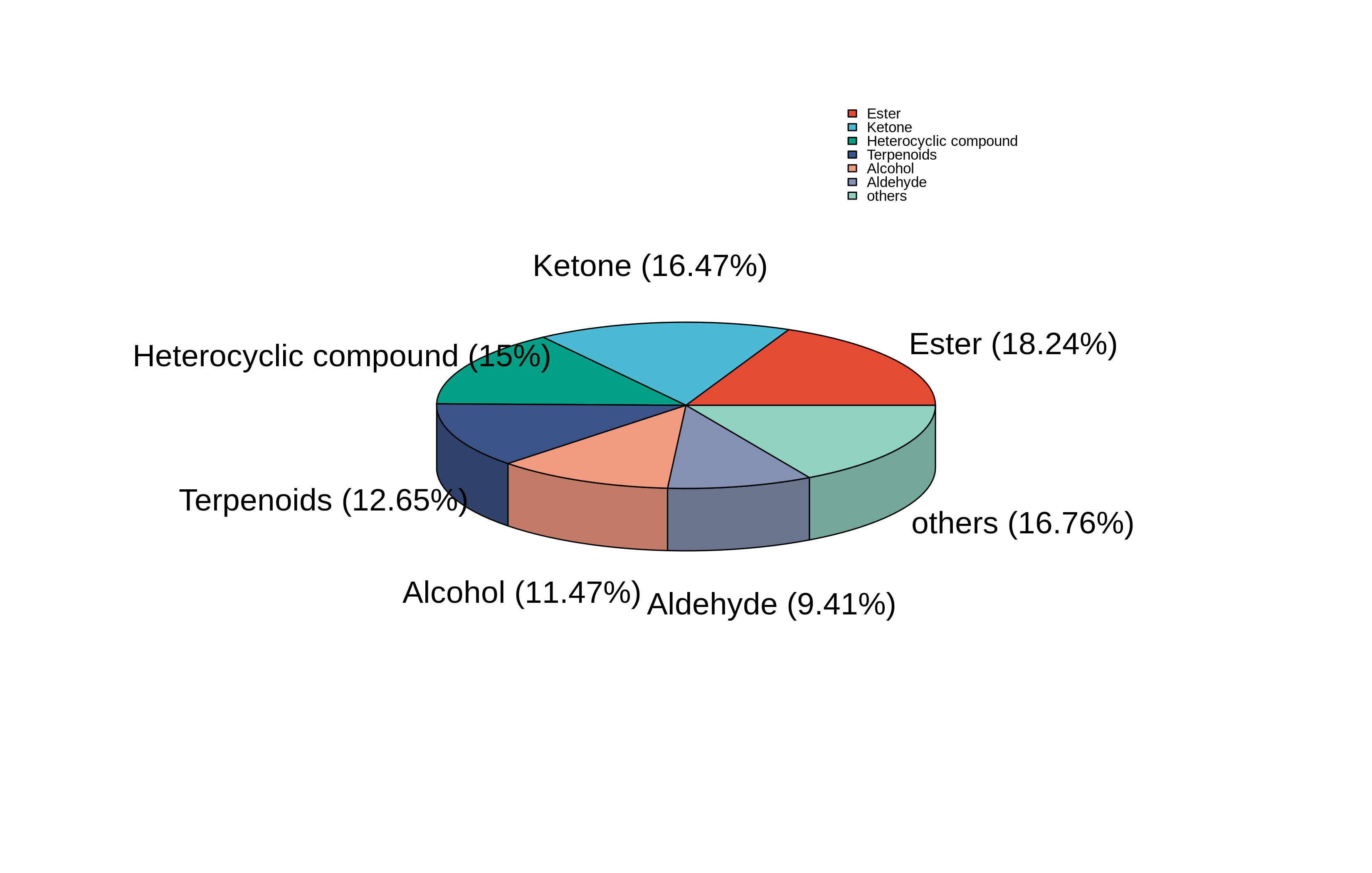

Supplement: Supplementary file 1 [file Image1.png]

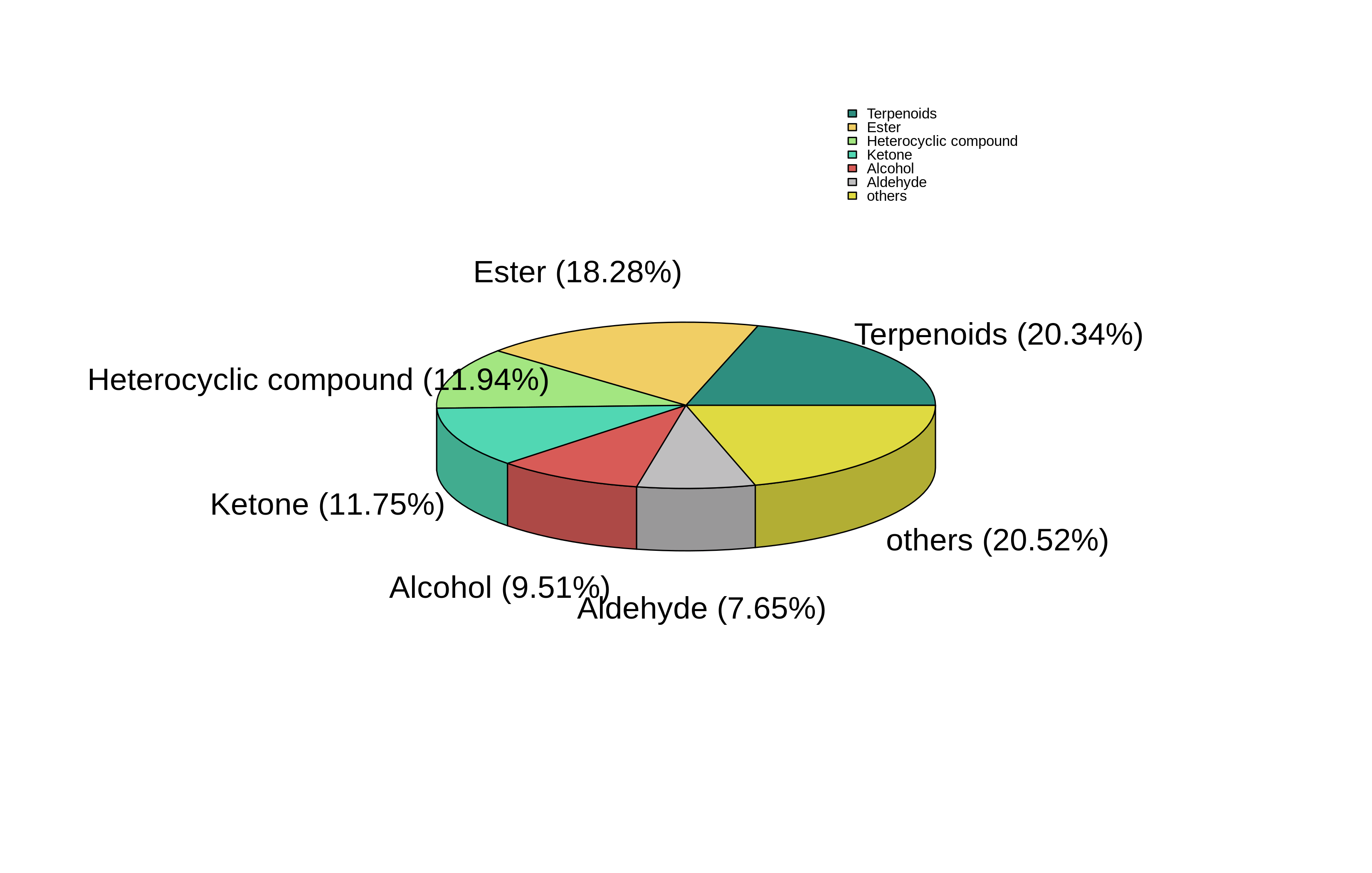

Supplement: Supplementary file 2 [file Image2.tiff]
